# The Signalling Role of the αvβ5-Integrin Can Impact the Efficacy of AAV in Retinal Gene Therapy

**DOI:** 10.3390/ph5050447

**Published:** 2012-05-02

**Authors:** Therese Cronin, Daniel C. Chung, Ying Yang, Emeline F. Nandrot, Jean Bennett

**Affiliations:** 1 F.M. Kirby Center for Molecular Ophthalmology, University of Pennsylvania, 422 Curie Blvd., Philadelphia, PA 19104, USA; Email: dcc2@mail.med.upenn.edu (D.C.C.); jebennet@mail.med.upenn.edu (J.B.); 2 INSERM, U968, 17 rue Moreau, Paris F-75012, France

**Keywords:** adeno-associated virus, retinal gene therapy, MFG-E8, αvβ5 integrin, photoreceptor outer segments

## Abstract

Sub-retinal injection of the common AAV2 pseudotypes frequently results in strong transduction of the retinal pigment epithelium (RPE) as well as the retina itself. This has been of benefit to date in human clinical trials using AAV, where the disease target is in the RPE. However, many mutations predisposing to retinal disease are located in the photoreceptor cells, present in the neural retina and not the RPE; in this case the sub-retinal injection route may cause an effective “loss” of therapeutic AAV to the RPE. The αvβ5 integrin receptor is highly expressed on the apical surface of the RPE, and is essential to the daily phagocytosis of the outer segment tips of photoreceptor cells. The transduction efficiency of AAV was tested in the retinas of β5^−/−^ mice lacking this receptor and showing defects in photoreceptor outer segment phagocytosis. Following sub-retinal injection of AAV2/5-eGFP, fluorescence was found to be stronger and more widespread in the neural retina of β5^−/−^ mice compared to wild-types with greatly reduced fluorescence in the RPE. Increased levels of the phagocytic signalling protein MFG-E8, the ligand for the αvβ5 integrin receptor, is found to have a moderate inhibitory effect on AAV transduction of the retina. However the opposite effect is found when only the integrin-binding domain of MFG-E8, the RGD (Arginine-Glycine-Aspartic acid) domain, was increased. In this case RGD enhanced AAV-mediated retinal transduction relative to RPE transduction. These results are presented for their relevance for the design of AAV-based retinal gene therapy strategies strategies targeting retinal/photoreceptor cells.

## 1. Introduction

**Figure 1:**
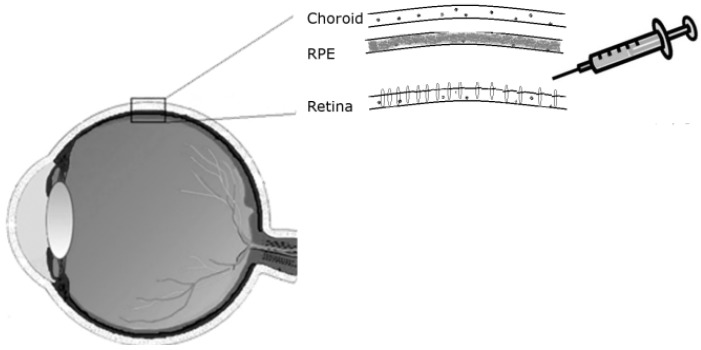
Subretinal injection route as used for delivery of therapeutic genes to the retina.

To date, the clinical success of retinal gene therapy has involved delivery of genes to the Retinal Pigment Epithelium (RPE) [1,2] which, though essential to the function and the maintenance of the retina is not part of the neural retina itself. It is in the photoreceptor cells of the neural retina that the early steps of light detection and signalling are carried out and photoreceptor-specific mutations result in rapid induction of apoptosis and loss of visual function [3]. It is probable that therapeutic strategies targeting photoreceptor-specific mutations, which lead to retinal degeneration, will require very high levels of therapeutic gene expression. At the current pre-clinical stage researchers are obliged to inject high titres of virus and the maximum volume possible into the confined subretinal space with no guarantee that the therapeutic level will be attainable. Adeno-Associated Virus (AAV) has proven useful for delivery of these therapies, however the pattern of expression for the serotypes that are commonly used demonstrate a bias toward RPE cell transduction following subretinal and even intravitreal injection [4]. There are some problems with this: efforts to fine-tune AAV-mediated gene expression for photoreceptors have been complicated by the significant “loss” of viral titre to the juxtaposed RPE cells (Figure 1).

Furthermore, where cell-specific promoters are used, the expression from the virus may be restricted to one cell type in the very heterogeneous cell populations of the retina, however the virus itself will nonetheless be present in non-targeted cells such as the easily transduced RPE cells—off-target effects as well as the unknown consequences of a high viral capsid load may be detrimental to these cells [5]. Finally, the production of such high viral titres is expensive in terms of time, money and space and limits the potential of smaller laboratories to carry out basic research using AAV as a delivery tool.

Despite the broad tissue tropism characteristic of AAV, transduction of the retina remains a challenge. We have found that AAV very effectively transduces the retinas of mice with a targeted deletion of a specific receptor involved in the synchronised diurnal uptake of the aged photoreceptor outer segments (POS). The integrin receptor αvβ5 normally abundant on the surface of the RPE is non-functional in β5^−/−^ mice leading to defective POS phagocytosis and decreased retinal adhesion [6]. These mice show greater ratio of retina/RPE AAV transduction compared to wild-type. We propose two potential reasons for this transduction bias: (1) The reduced level of retinal adhesion and impaired phagocytosis in the β5^−/−^ retina may lead to the retention of the AAV particle in the photoreceptor cell; (2) the loss of a potential coreceptor in, the αvβ5 integrin, normally abundant on the RPE surface, may increase the utilisation of alternative coreceptors by AAV, these other coreceptors, may be potentially more prominent on the retinal side; (3) the decreased level of adhesion between the neural retina and RPE of β5^−/−^ mice may allow for greater retinal detachment upon subretinal injection and greater AAV uptake. This may be due to the reduced level of rhythmic phagocytosis that occurs in this model leading to the retention of the AAV particle in the photoreceptor cell. Should POS phagocytosis impact the level of AAV transduction than it was expected that we would see changes in AAV-mediated expression (in this case and luciferase) at times of increased phagocytosis by the RPE. We investigate these two possibilities further in wild-type (WT) mice by using the endogenous signalling ligand molecule for the αvβ5 receptor and consider its implications for the clinical use of AAV.

## 2. Results and Discussion

### 2.1. AAV2/5 Transduction of the β5^−/−^ Mouse Retina

Drugs can be delivered to the retina by injecting them in solution between the retina and the RPE, hence called sub-retinal injection. For administration of treatments targeting the retina’s outer nuclear layer, a sub-retinal mode of delivery is preferable to an intravitreal route, as it allows direct contact with the photoreceptor cells (Figure 1).

**Figure 2:**
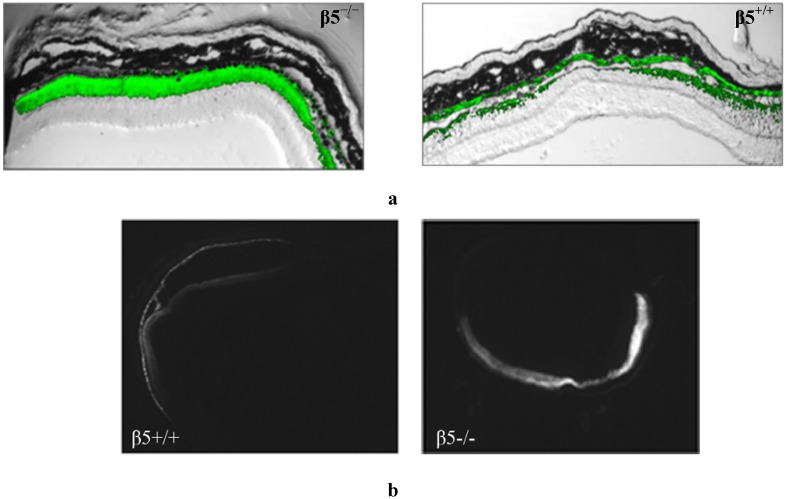
Frozen retinal sections taken from mice (n = 10), 5 weeks after sub-retinal injection with AAV2/5(CMV-eGFP). (**a**) The representative level of fluorescence in a mouse that has no functional expression of the β5 integrinreceptors (β5^−/−^) compared to WT (β5^+/+^) are shown at 20X magnification; (**b**) Comparable nomarski full-field images are shown in 4× magnification.

Following subretinal injection the therapeutic solution is expected to be equally distributed between the RPE and the neural retina. However, a therapy that is delivered by an AAV vector may show a preference for RPE transduction, depending in particular on the serotype used. The β5^−/−^ mouse model does not express functional αvβ5R and shows some defects in the adhesion of the retina to the RPE as well as a marked loss of the circadian peak of phagocytosis and engulfment of shed POS [7,8] We examined the transduction pattern of a CMV-driven fluorescent eGFP marker from AAV2/5 following subretinal injection in β5^−/−^ mice. In contrast to that observed in WT mice, the injected β5^−/−^ mice show a stronger (Figure 2a) and more widespread (Figure 2b) level of retinal fluorescence combined with a reduced level of RPE fluorescence as evident in horizontal depth-matched frozen sections.

It is inferred that the absence of αvβ5 integrin function is responsible for the altered viral transduction pattern. This loss has both signalling and mechanical consequences for the retina and either or both may be affecting AAV transduction—the loss of αvβ5 receptor function may lessen the possibility of AAV particles reaching the RPE as POS-cargo. At the same time the reduced retinal/RPE adhesion may lead to greater retinal detachment upon subretinal injection. However, while this latter physical change accounts for more viral delivery overall it does not explain why there is greater transduction of retina compared to RPE in β5^−/−^ mice. We therefore focus our investigation of this retina/RPE bias on the signalling effects. We propose that the β5^−/−^ mice may exhibit a skewed AAV retinal/RPE transduction due to changes in subretinal phagocytic signalling and/or due to loss of a putative AAV coreceptor, the β5 integrin.

### 2.2. Increased Phagocytic Signalling Alters the AAV Transduction Profile Following Subretinal Injection in Wild-Type Mice

The RPE is generally very effectively transduced following subretinal injection of AAV. This is because it is exposed on the apical side to the subretinal space and can receive its share of virus following injection. In addition, it is possible that an AAV having successfully transduced a photoreceptor cell may be lost in shed outer segments before it can be trafficked to the photoreceptor nucleus. Following uptake of these AAV-carrying segments by the RPE, the AAV can escape the lysosomal degradation pathway and be expressed by the RPE cell. Fundamental to the uptake of POS by RPE is the protein Milk Fat Globule-EGF8 (MFG-E8) protein ligand, which binds to apoptotic cells and facilitates their removal through bridging interaction with phagocytes. As well as stimulating phagocytosis the MFG-E8/integrin interaction may havehas important adhesive effects [9].

**Figure 3:**
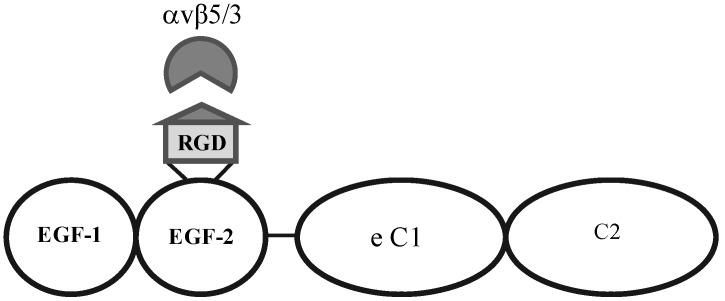
Structural motifs of MFG-E8, containing two EGF-like domains, the second of which carries the integrin-binding motif. This protein is the signalling ligand for the αvβ5-integrin receptor in the retina.

**Figure 4:**
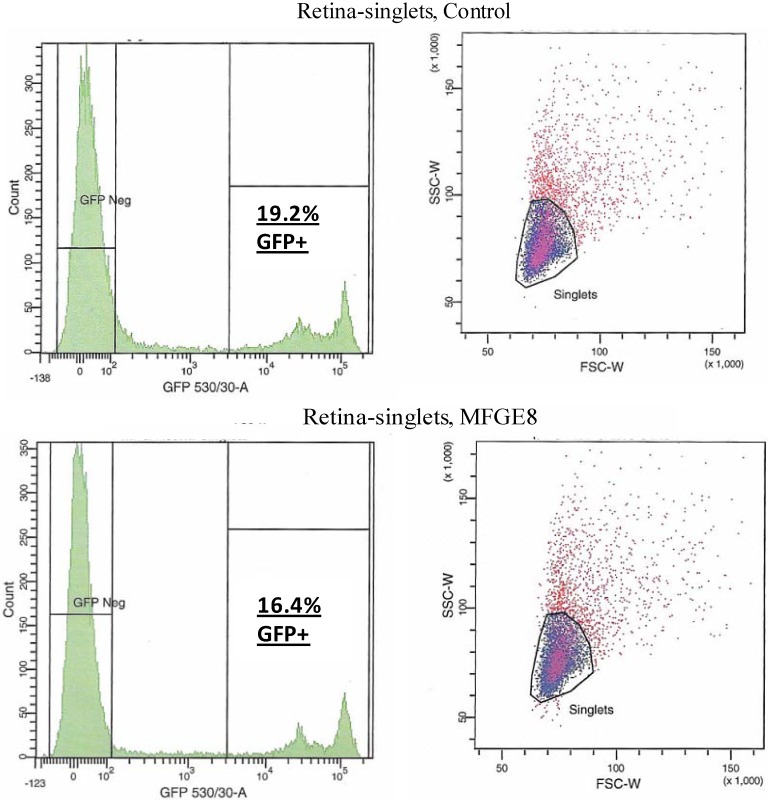
FACS analysis was used to quantify the level of transduction achieved by AAV in the presence of MFG-E8. Four weeks following co-subretinal injection of MFG-E8 with AAV2/5(eGFP) in adult mice (n = 10), retinas were dissociated and cells counted. 19.2% of retinal cells are positive for eGFP in the control PBS pool compared to 16.4% eGFP-positive retinal cells in the test MFG-E8 pool.

Nandrot *et al*. have shown secreted MFG-E8, which localizes to the subretinal space, to be the primary ligand responsible for stimulating synchronized αvβ5 integrin signalling and specific RPE phagocytosis in the mouse retina [9,10]. We wished to investigate whether increased levels of this molecule in the subretinal space would lead to more AAV being ‘redistributed’ to the RPE due to an increase in αvβ5 signalling and POS uptake*.* A 0.3 mg/mL solution of MFG-E8 (Figure 3) was co-subretinally-injected with AAVs expressing either eGFP (for FACS analysis) or the firefly luciferase gene (for plate-reader analysis) into WT mice. The contralateral eye was injected with PBS and the AAV. By FACS analysis, a modest decrease in the number of fluorescent retinal cells was counted when the αvβ5 ligand MFG-E8, was injected with the viral vector: 16.4% of cells from the MFG-E8 retinas compared to 19.2% from the control retinas (Figure 4 and Table 1).

**Table 1:** Cell numbers from FACS analysis of AAV subretinal transduction with increased levels of MFG-E8. The number of cells that are transduced (GFP +ve) or not (GFP −ve) by virus expressing eGFP are shown.

Population	# Events	% Total
	*Retina, Control*	
All events	20,000	
Live singlet cells	9,344	100
GFP −ve	6,840	34.2
GFP +ve	1,792	19.2
	*Retina, MFG-E8*	
All events	20,000	
Live singlet cells	8,683	100
GFP −ve	6,871	34.4
GFP +ve	1,420	16.4

**Figure 5:**
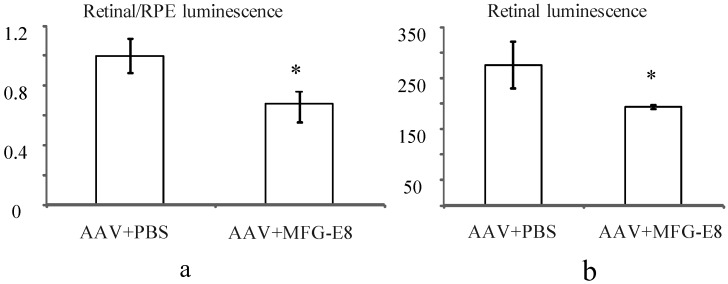
(**a**) Effect of MFG-E8 on the luminescence profile (retinal luminescence)/(RPE luminescence), following co-subretinal injection with AAV-luciferase in adult mice (n = 9), *p* = 0.03. Overall the ratio of retina to RPE transduction is reduced when MFG-E8 is present in the viral adjuvant. The retinal luminescence (**b**) shows the absolute reduction in retinal transduction due to MFG-E8, *p* = 0.04.

In order to account for potential variability in injection between mice, the transduction ratio for an AAV expressing firefly luciferase, (retina luminescence/RPE luminescence), was tested. An AAV vector expressing firefly luciferase was coinjected with PBS or MFG-E8 in the alternate eyes of nine CD1 mice. This outbred albino strain was used as they have no pigment in the RPE, which may otherwise prevent luminescence readings from the RPE fraction. Moreover they do not have a retinal degeneration (*rd*) mutation as do many other outbred mouse strains. A luciferase assay was used to determine the relative luminescence of an RPE fraction compared to a retinal fraction from each individual mouse and the average ratios plotted (Figure 5a). An average ratio of 1 was determined for the AAV-luciferase + PBS injected mice. However when the MFG-E8 ligand was coinjected with the luciferase-expressing AAV, the retina/RPE transduction ratio dropped to 0.68 (*p* = 0.03, Figure 5a). Similarly, the absolute retinal levels of luciferase expression also show decreased AAV transduction when coinjected with MFG-E8 (Figure 5b) suggesting an RPE bias for transduction when MFG-E8 level was increased in the subretinal space. This supports the hypothesis that triggering αvβ5-integrin-induced phagocytosis may reduce the level of AAV-mediated expression in the neural retina.

### 2.3. Blocking the αvβ5 Integrin Coreceptor Alters AAV Uptake Following Subretinal Injection in Wild-Type Mice

The broad tissue tropism characteristic of AAV may be attributed to the range of ubiquitously expressed receptors and coreceptors which the virus can recruit for transduction. Heparin Sulfate Proteoglycan (HSPG) was originally identified as the primary attachment receptor of AAV2 [11], since then several coreceptors necessary for cell entry have been identified. Amongst these, are the integrins which have been found to be involved in the attachment, entry and uncoating of AAV [11]. In addition to important roles in cell-cell adhesion and cell signalling the αvβ5 integrin receptor has been shown to serve as a coreceptor for adenovirus and has been suggested to serve as coreceptor for some serotypes of AAV, in particular for AAV serotype 1 [12]. It is a possibility that the absence of expression of the αvβ5 integrin on the RPE apical surface of the β5^−/−^ mouse may increase the utilisation of alternative coreceptors by the virus, such as FGFR1, HGF and PDGF, which may be more prominently expressed on the retinal side. MFG-E8 can serve as a bridging protein via its Arginine-Glycine-Aspartic Acid (RGD) motif between αvβ5 integrin receptors at the RPE surface and spent POS in the subretinal space [13]. The RGD domain of the adenovirus is shown to interact with the integrin receptor and for this reason we tested whether the RGD peptide may be used to saturate the receptor in WT mice. This would have the effect of effectively blocking transduction of the RPE at times of AAV injection thus redirecting the virus to alternative receptors on the adjacent retinal cells. In theory this should lead to similar transduction characteristics as those of the β5^−/−^ mouse. AAV-eGFP was cosubretinally injected with RGD peptide in one eye and a control RAD peptide in the contralateral eye of adult WT mice. After 5 weeks the mice were euthanized and retinal fluorescence levels compared by FACS analysis (n = 10).

**Figure 6:**
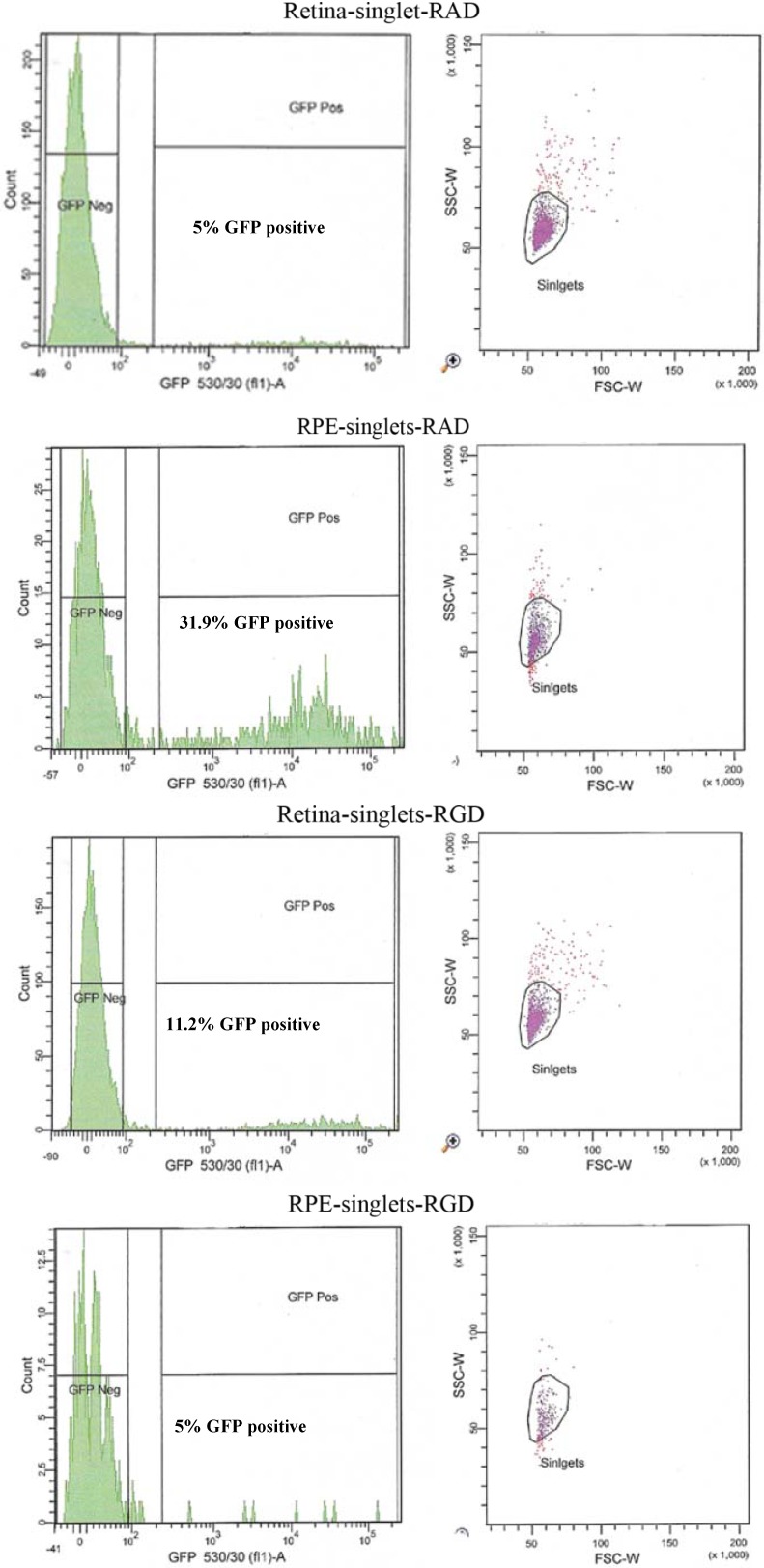
FACS analysis of the effect of peptides on the cellular transduction pattern following co-subretinal injection with AAV2/5 (CMV-eGFP) in adult mice. Only 5% of retinal cells coinjected with RAD peptide control are positive for eGFP compared to 11.2% of eGFP-positive retinal cells coinjected with RGD. By contrast RPE cells from RAD-injected mice are 31.9% positive for eGFP compared to only 2.7% eGFP-positive RPE cells in the RGD pool.

An altered pattern of transduction was found in the cell counts of the dissociated RPE and retinal cells from 10 eyes used for FACS analysis. In fact, total transduction was reduced for RGD-injected versus RAD-injected retinas; however, the retinal-specific eGFP expression of the RGD cells was increased 4.8-fold while the RPE-specific eGFP expression of RGD cells was reduced by approximately 6-fold (Figure 6 and Table 2). In order to verify the extent to which this RPE “blocking” by RGD peptide might enhance retinal transduction by AAV, we used an AAV expressing the firefly luciferase gene for injection into albino CD1 mouse eyes which lack of pigment in the RPE facilitating the luminescence readouts from the RPE cell lysates.

**Table 2:** Cell numbers from FACS analysis of AAV subretinal transduction with increased levels of MFG-E8. The number of cells that are transduced (GFP +ve) or not (GFP –ve) by virus expressing eGFP are shown.

Population	# Events	% Total	# Events	% Total
	***Retina RAD***		***RPE RAD***	
Live singlet cells	4,297	100	864	100
GFP −ve	4,035	93.9	562	65
GFP +ve	215	5	276	31.9
	***Retina RGD***		***RPE RGD***	
Live singlet cells	3,743	100	314	100
GFP −ve	3,219	86	298	95
GFP +ve	429	11	8	5

**Figure 7:**
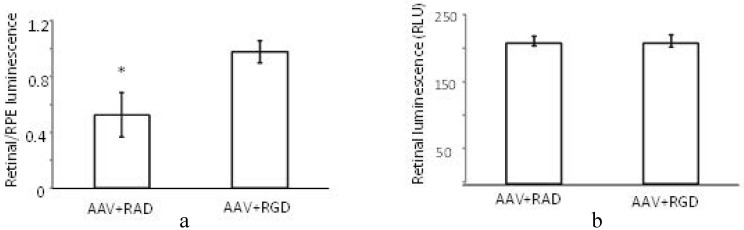
Effect of peptides on luminescence profile following co-subretinal injection with AAV2/1(CMV-luciferase) in WT mice (n = 8). While the ratio (retinal luminescence)/(RPE luminscence) is higher for RGD co-injected mice, (**a**, *p* = 0.017), the absolute retinal luminescence (**b**) suggests that the RPE-blocking effect is not improving retinal transduction.

The (retinal luminescence)/(RPE luminescence) ratio is significantly higher where AAV (CMV-luciferase) was coinjected with RGD peptide compared to the control RAD (Figure 7a). This implies that the RPE-bias evident in the control injection virus is less pronounced in the presence of the αvβ5 integrin blocking RGD peptide. As we are interested in the ratio of AAV mediated expression in the retina versus the RPE the variability in the success of subretinal injections is of less concern. However, it is nonetheless worth noting that the absolute levels of luminescence in the retina are found to be equivalent between RAD and RGD retinas (Figure 7b). Thus, through FACS analysis and luciferase reporter assay we have determined that the RGD domain alone can block the RPE uptake, presumably through saturation of the integrin receptor used for cell entry*.* However in contrast to the FACS study, the luciferase assay does suggest that this blocking effect may not necessarily enhance retinal transduction. This would imply that the viral transduction pattern of the β5^−/−^ mouse is not due to virus/receptor interactions.

## 3. Experimental

### 3.1. Mouse Models

C57Bl/6 C57BL/6(breeding stock from Jackson Laboratories, Bar Harbor, ME, USA) and CD1 mice were housed in the Laboratory Animal Stemmler Facility at the University of Philadelphia. These mice were used at 5 weeks and 10 weeks of age for FACS analysis (n = 10) and cryosectioning (n = 4), and luciferase assay (n = 9). The β5^−/−^ mice [1] were housed at Animal Facility of Hospital St. Antoine (UPMC, Paris, France) were treated at 10 weeks of age and analysed by cryosectioning (n = 10). All mice were maintained in sterilized plastic microisolator cages and given sterilized standard laboratory chow and tap water *ad libitum*. All experimental protocols were reviewed and approved following the guidelines set forth in the National Institutes of Health “Guide for Care and Use of Laboratory Animals”.

### 3.2. AAV Vectors and Reagents

rAAV2/5-eGFP and rAAV2/1-eGFP vector lots were produced by the Center for Cellular and Molecular Therapeutics at The Children's Hospital of Philadelphia (Philadelphia, PA, USA). Recombinant mouse MFG-E8 was ordered from R&D systems (Minneapolis, MN, USA) and reconstituted at 100 μg/mL in sterile PBS. 2 µL of a 1:1 mix of this peptide solution and the viral solution was used for subretinal injection. The RAD and RGD peptides, used in both linear (H-Arg-Xxx-Asp-OH) and cyclic (Arg-Xxx-Asp-D-Phe-Val) conformations, were ordered from Bachem Inc. (Torrance, CA, USA). These were reconstituted to 1 mg/mL in sterile PBS. 2 µL of a 1:1 mix of this peptide solution and the viral solution was used for subretinal injection.

### 3.3. Subretinal Injection

Adult C57Bl/6 mice were anesthetized with inhaled isoflurane and the right eye injected with 2 μL of MFG-E8/AAV mixture (0.3 mg/mL solution in PBS) and the left eye with 2 μL of PBS. For subretinal injection in the right eye, the animal was placed on the left lateral side. One drop of 1% proparacaine instilled into the right eye. The temporal conjunctiva was grasped 1 mm posterior to the limbus and the eye rotated nasally. With the eye in a nasal position, a small peritomy was made with Vannas curved scissors. The underlying tenon’s capsule was incised, to expose bare sclera. A straight 30 gauge needle was used to make a small sclerotomy 3 mm posterior to the limbus, with a depth of 1 to 1½ mm. Scleral vessels were avoided to prevent bleeding. The vector was drawn in a 10 μL Hamilton syringe with a 33 gauge needle. The needle tip penetrated the sclerotomy site for 1 mm in depth, at which point 2 μL of the vector was injected into the sub-retinal space. Similar procedure was done on the contralateral side. Animals were placed on a heated pad and observed until full recovery from the anesthesia, and analgesia given as needed.

### 3.4. Immunohistochemistry

Mice were euthanized by CO_2_ and rapid cervical dislocation. The eyes were immersion-fixed in 4% paraformaldehyde overnight. They were then placed in 10% sucrose solution followed by 20% sucrose (for 1 h each) and finally 30% sucrose overnight at 4 °C. They were embedded in cryogel (Triangle Biomedical Sciences Inc, Durham NC, USA), and snap frozen. 10 μM sections were made using a Leica CM1850 cryostat.

### 3.5. FACS Analysis

Eyes used for cell counting were dissected in sterile PBS, prewarmed to 37 °C to ensure separation of choroid/RPE and neural retina. The RPE and retinal fractions were dissociated separately using papain supplemented with DNAse1 and the protocol as outlined by the Worthington dissociation system used (Worthington Biochemical Corp., NJ, USA).

### 3.6. Luciferase Assay

Eight CD1 mice were co-injected with a 1:1 mix of 1 µL of AAV2.1-luciferase and MFG-E8 (R&D systems, MN, USA) or PBS. Three weeks after injection, the mice were sacrificed and retinas and RPE dissected separately in warmed HBSS. The tissue was snap-frozen in liquid nitrogen and stored at −80 °C prior to assay. The frozen tissue was grinded with a pestle and resuspended in 100 µL of 1× lysis buffer (Promega, CA, USA). The debris was pelleted by centrifugation and 20 µL of the supernatant aliquoted in triplicate into the wells of a 96-well white-walled microtiter plate. 100 µL of luciferase assay reagent (Promega, CA, USA) was added to each well by an automated Wallac Victor plate reader (Perkin Elmer Waltham, MA, USA) and readings recorded. The luminescence ratio of retina/RPE was determined for each eye and the average computed.

### 3.7. Statistics

Standard *t*-tests were used to determine significance in differences between pairs. For all experiments data were expressed as the mean ± the Standard Error of the Mean (SEM). In the figures, different levels of significance are indicated by * if *p* < 0.05, ** *p* < 0.01.

## 4. Conclusions

We show here increased retinal transduction by AAV in β5^−/−^ mice. We investigate the causes for this, particularly in light of the implications they may have for AAV-mediated retinal gene therapy. Two possible causes were examined: (1) the impact of the αvβ5 integrin signalling molecule, MFG-E8 on AAV retinal transduction and (2) the putative coreceptor role of the RPE-abundant αvβ5 integrin for AAV uptake.

We find here that MFG-E8 appears to increase the AAV transduction of RPE relative to the retina, an effect that may relate to the phagocytic signalling induced by this ligand on the RPE cells. It is possible that MFG-E8 may enhance viral uptake in the RPE due to the increase shedding of POS that “carry” AAV to the RPE. The implication being that AAV retinal delivery strategies should take into account the timing of AAV injections relative to light-onset and innate circadian cues. This may include alternative time-points of injection and light-restriction regimens that may alter the POS trafficking in the subretinal space. The alternative possibility, that αvβ5 integrin abundant on the RPE, may serve as a coreceptor for AAV—was investigated by RGD-blocking experiments. However while AAV “loss” to the RPE is reduced we do not always find that this blocking strategy increases the titre reaching the retina. Moreover, the role αvβ5 integrin plays in AAV uptake is still speculative, based largely on its interaction with adenovirus [12]. Therefore the altered profile of AAV transduction in the β5^−/−^ retina may not necessarily be due to virus/receptor interaction. Instead it may be linked to its role in stimulating intracellular internalisation pathways and have an inhibition effect—RGD may be blocking αvβ5-linked activation of intracellular pathways needed for POS uptake [6].

It remains possible that the increased retinal transduction is caused by the loss of adhesion and therefore the more significant retinal detachment that might arise upon subretinal injection of the β5^−/−^ mouse. However this does not account for the reduced transduction of the RPE that appears in parallel with the improved neural retinal transduction. It is important to determine how such a viral transduction profile might arise as it may allow for further optimisation of gene therapy strategies that target photoreceptor-based diseases.
